# Radiation-Induced Soft Tissue Injuries in Patients With Advanced Mandibular Osteoradionecrosis: A Preliminary Evaluation and Management of Various Soft Tissue Problems Around Radiation-Induced Osteonecrosis Lesions

**DOI:** 10.3389/fonc.2021.641061

**Published:** 2021-04-28

**Authors:** Chunyue Ma, Weijin Gao, Zhonglong Liu, Dan Zhu, Fengshuo Zhu, Xiaoguang Li, Yue He

**Affiliations:** ^1^ Department of Oral Maxillofacial-Head and Neck Oncology, Shanghai Ninth People’s Hospital, School of Medicine, Shanghai Jiao Tong University, Shanghai, China; ^2^ Department of Oral and Maxillofacial Surgery, First Affiliated Hospital of Wenzhou Medical University, Wenzhou, China; ^3^ Department of Radiology, Shanghai Ninth People’s Hospital, School of Medicine, Shanghai Jiao Tong University, Shanghai, China

**Keywords:** osteoradionecrosis, soft tissue injury, toxicity, fibrosis, evaluation, management, correlation, risk

## Abstract

**Objectives:**

Radiation-induced soft-tissue injuries (STIs) in mandibular osteoradionecrosis (ORN) are not well studied regarding their correlations with nearby bone lesions. The aim of this study is to investigate the severity of radiation-induced STIs in advanced mandibular ORN and its relationship with hard-tissue damage and postoperative outcomes.

**Methods:**

A retrospective study was performed in our institution from January 2017 to December 2019. Aside from demographic factors, the associations between the triad ORN variables (irradiation doses, ORN stages, ORN sizes) and radiation-related STI factors, vascular characteristics, and postoperative functional recovery were assessed. In addition, the severity of STI was also compared with treatment outcomes. Such correlations were established *via* both univariate and multivariable analyses.

**Results:**

A total number of 47 patients were included. The median follow-up reached 27 months. Nasopharyngeal cancer was the histology type among most patients (n = 21, 44.7%). The median irradiation doses reached 62 Gy (range, 40–110 Gy). For STI, the symptom scoring equaled an average of 5.4 (range from 1 to 12), indicative of the severity of STI problems. During preoperative MRI examinations, signs of hypertrophy or edema (n = 41, 87.2%) were frequently discerned. Most patients (n = 23, 48.9%) also had extensive muscular fibrosis and infection, which required further debridement and scar release. Surprisingly, most STI factors, except cervical fibrosis (p = 0.02), were not in parallel with the ORN levels. Even the intraoperative soft-tissue defect changes could not be extrapolated by the extent of ORN damage (p = 0.096). Regarding the outcomes, a low recurrence rate (n = 3, 6.9%) was reported. In terms of soft tissue-related factors, we found a strong correlation (p = 0.004) between symptom scores and recurrence. In addition, when taking trismus into consideration, both improvements in mouth-opening distance (p < 0.001) and facial contour changes (p = 0.004) were adversely affected. Correlations were also observed between the intraoperative soft-tissue defect changes and complications (p = 0.024), indicative of the importance of STI evaluation and management.

**Conclusions:**

The coexistence of hard- and soft-tissue damage in radiation-induced advanced mandibular ORN patients reminds surgeons of the significance in assessing both aspects. It is necessary to take the same active measures to evaluate and repair both severe STIs and ORN bone lesions.

## Introduction

Treatment of advanced head and neck malignancies primarily involves radiotherapy and chemotherapy with the goal of improved survival outcomes (1, 2). While highly effective in some cases, especially those with nasopharyngeal or oropharyngeal cancers, radiation therapy can cause a multitude of chronic complications, among which osteoradionecrosis (ORN) is one of the most devastating (3). ORN of the jaw has long been characterized by necrotic bone exposure (4, 5). Despite the recent change in the definition for additional soft tissue considerations, most studies regarding ORN evaluations still focus on the simple elements of osseous injuries (5, 6). Based on the classic theory of pathogenesis, radio-induced fibrosis can also occur in soft tissues due to “hypoxia, hypovascularization, and hypocellularity” in the surrounding cellular matrix (7). As proof of such theory, radiation-induced symptoms, such as swelling, dysphagia and trismus, were also frequently observed in advanced ORN cases (8). Nevertheless, most recent studies have focused solely on necrotic bone management (5, 9). Therefore, reports regarding the incidence and severity of such soft tissue problems, let alone management, are scarce. In view of the status quo, we intended to investigate the severity of soft-tissue injuries (STIs) in patients diagnosed with advanced mandibular ORN, which has been largely overlooked in the literature. The focus of our study was on triad dimensions regarding STI evaluation and bone injury relations, STI management, and prognosis and predictions after STI debridement.

## Materials and Methods

### Study Population and Inclusion Criteria

With ethical approval from the Institutional Ethics Committee, we retrospectively reviewed and collected anonymized clinical information regarding patients with advanced mandibular osteoradionecrosis who had received surgical treatment in our institution from January 2017 to December 2019. The definition of “advanced osteoradionecrosis” was based on the Bone-Soft (BS) tissue staging system (10) (**Supplementary Tables 1** and **2**). According to the focus of the study, the inclusion criteria were as follows: 1) patients diagnosed with BS stage II-III diseases who received surgical debridement and segmental mandibulectomy; 2) patients without synchronous locoregional recurrences of malignancies or second primary or radio-induced malignancies; 3) those with complete records of preoperative computed tomography (CT), magnetic resonance imaging (MRI) and panoramic X-ray examinations; 4) patients with follow-up and functional information; and 5) patients who also gave written consent for the study.

### Demographic Information and Medical Histories

The demographic information was directly collected from the hospital chart database. Clinical data, specifically, medical (surgical) treatment histories, comorbidities, prior radiation dosages, adjuvant therapies, prior conservative hyperbaric oxygen (HBO) treatment, current ORN stages, and affected mandibular subsites were also reviewed and compared.

### Hard- and Soft-Tissue Evaluations

**Figure 1:**
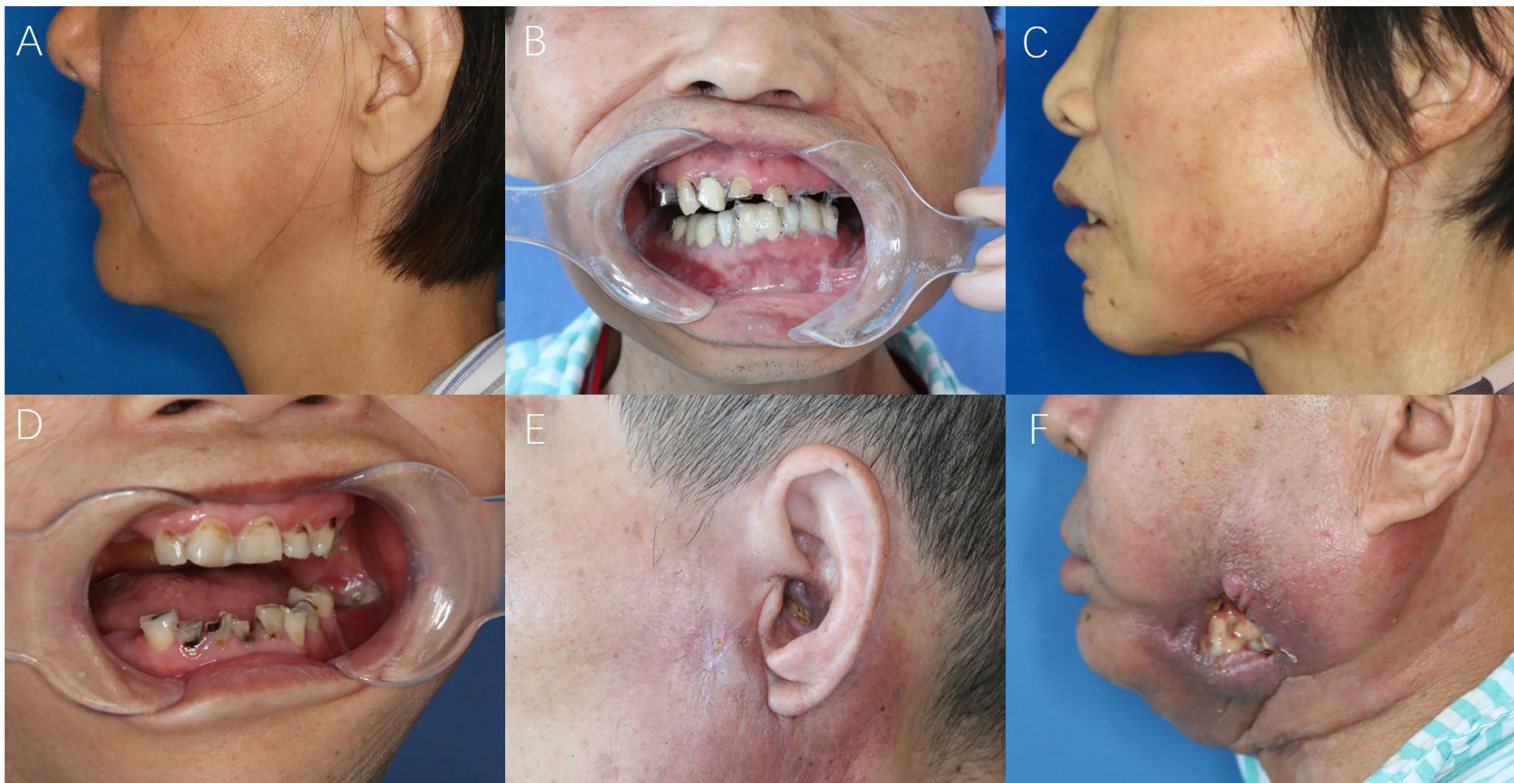
The symptom-based scoring system for STI evaluations. **(A)** Stiffness of masseter or cervical muscles; **(B)** Difficulty in mouth-opening (trismus); **(C)** Swelling and skin discolor; **(D)** Intraoral mucosal fistula; **(E)** Extraoral cutaneous fistula; **(F)** Large oro-cutaneous fistula with persistent suppuration.

**Figure 2:**
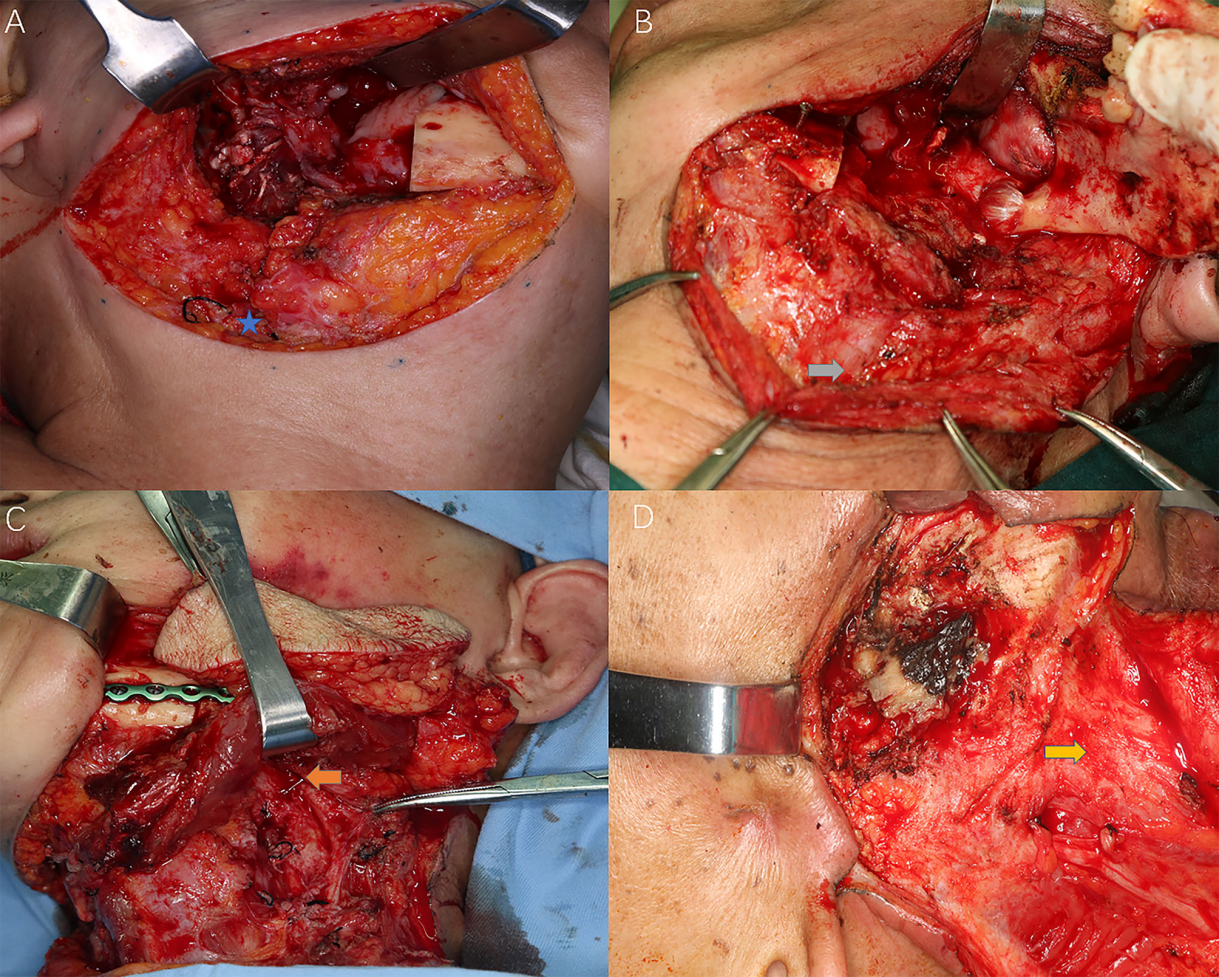
Different levels of cervical fibrosis found intraoperatively for the reflection of radiation-induced STIs. Blue star: external jugular vein (EJV); Grey arrow: sternocleidomastoid (SCM) muscle fibrosis and EJV stenosis; Orange arrow: stenosis of facial artery; Yellow arrow: frozen neck with inseparable fibrotic cervical sheath. **(A)** Slight subcutaneous fibrosis without external jugular vein stenosis; **(B)** Intermediate muscular fibrosis [sternocleidomastoid muscle (SCM)] with external jugular vein stenosis; **(C)** Severe fibrosis with both SCM and superficial artery (facial artery) stenosis; **(D)** Frozen neck with inseparable fibrotic internal jugular vein or cervical sheath.

**Figure 3:**
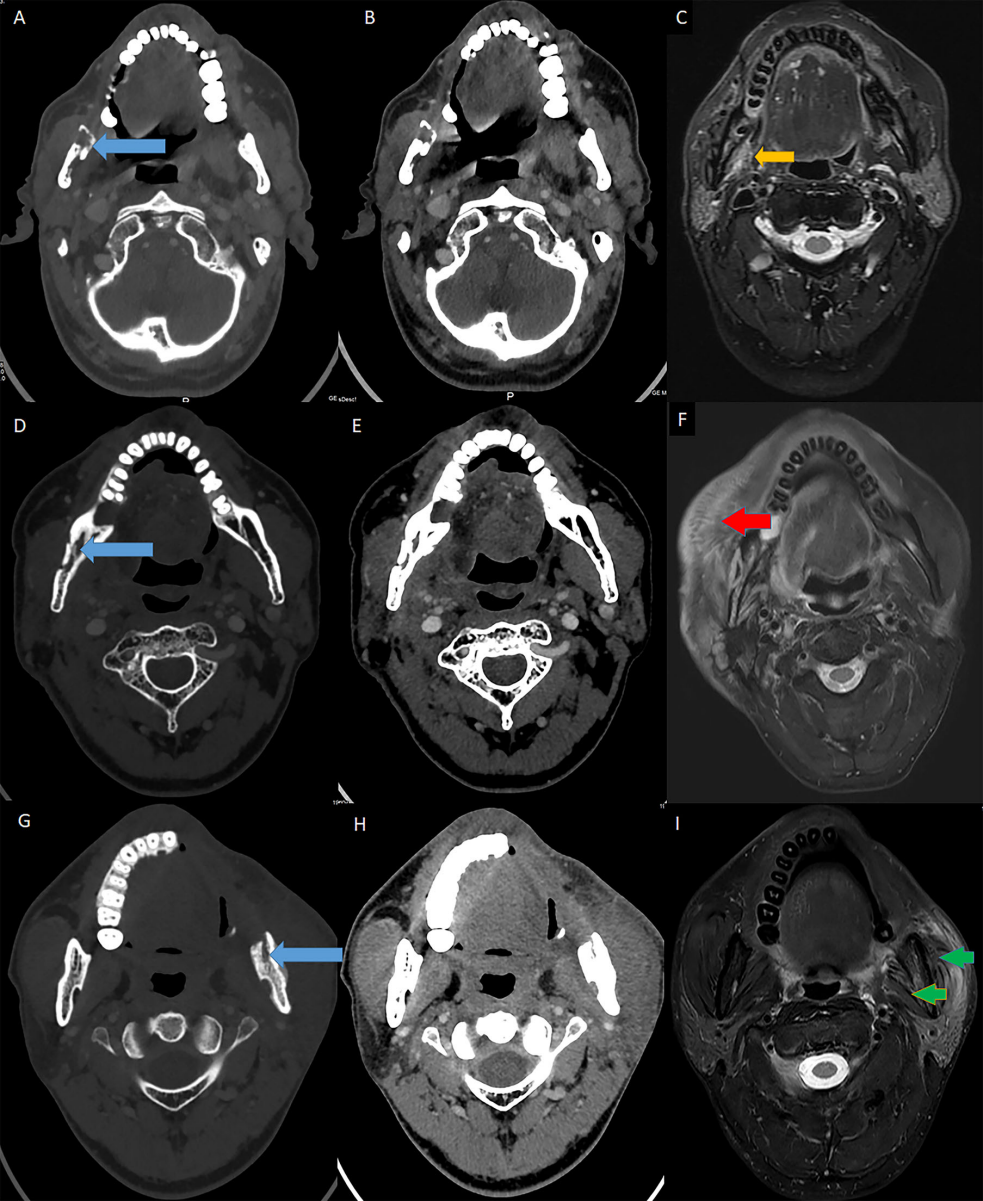
MRI evidence for muscular STI in ORN patients. Blue arrow: ORN lesions; Orange arrow: muscular hypertrophy; Red arrow: muscular edema; Green arrow: muscular atrophy. **(A)** The axial enhanced CT (bone window) showed the ORN lesion in the ramus. **(B)** The axial enhanced CT (soft-tissue window) revealed both the ORN and soft tissue content. **(C)** The axial T2-weighted MRI showed hypertrophy in the pterygoid muscles due to STI. **(D)** The axial enhanced CT (bone window) showed the ORN lesion in the body and ramus (the second patient). **(E)** The axial enhanced CT (soft-tissue window) revealed both the ORN and soft tissue content (the second patient). **(F)** The axial T2-weighted MRI showed edema in the ipsilateral masseter muscles due to STI (the second patient). **(G)** The axial enhanced CT (bone window) showed the ORN lesion in the ramus (the third patient). **(H)** The axial enhanced CT (soft-tissue window) revealed both the ORN and soft tissue content (the third patient). **(I)** The axial T2-weighted MRI showed atrophy in both the pterygoid and masseter muscles due to STI (the third patient).

Detailed characteristics regarding bone and soft tissue involvement were obtained by analyzing the clinical and radiographic records. First, to clearly delineate radio-induced hard tissue injuries, necrotic bone information was presented by the subsites of mandibular ORN (ipsilateral or bilateral; body or ramus) with areas of radiolucency with sclerotic changes in CT scans. Furthermore, types of intraoperative bone defects, according to Brown’s classification (11), were also recorded by reviewing surgical charts. In addition, the severity of STI was assessed by dichotomized (subjective and objective) methods. Within the subjective soft-tissue evaluations, a symptom-based scoring system was tentatively developed to simplify the multitude and scale of discomfort reported in the presurgical consultation records. Stiffness of masseter or cervical muscles as 1; difficulty in mouth-opening as 2; swelling and skin discolor as 3; intraoral mucosal defect as 4; extraoral or oro-cutaneous fistula as 5; fistula with persistent suppuration as 6 (**Figure 1**). The final score of this symptom-based system was the addition of these scores. In addition, another subjective evaluation of STI was determined by the intraoperative debridement of ORN-involved local structures: involving only mucosal and submucosal tissues as 1; involving both cutaneous and mucosal tissues as 2; involving masseter muscle as 3; and involving other muscles as 4. Cervical fibrosis was also analyzed with intraoperative descriptions for indirect reflection of the radio-induced STIs: 1 as slight subcutaneous fibrosis without external jugular vein stenosis; 2 as intermediate muscular fibrosis [sternocleidomastoid muscle (SCM)] with external jugular vein stenosis; 3 as severe fibrosis with both SCM and superficial artery (facial artery) stenosis; and 4 as frozen neck with inseparable fibrotic internal jugular vein or cervical sheath (**Figure 2**). The objective assessment of local STIs was mainly based on radiographic evidence. First, different extents of osseous changes, such as osteolytic cortical erosion involving a single buccal or lingual surface, cortical erosion involving bicortical surfaces, bone fragmentation or sequestrum, or even bone fracture, were detected on CT, while neighboring STI changes, such as hypertrophy, atrophy, and edema were also found on MRI. The specific features for these STI changes were defined according to Marieke’s criteria (12). Specifically, muscular hypertrophy or atrophy was evaluated on both T2- and T1-weighted MRI images. Loss of muscle volume and fatty changes within the muscle were regarded as signs of atrophy, whereas an increased volume of muscle represented hypertrophy. Edema of the masticatory muscles (attached to mandible) was basically evaluated on T2-weighted MRI images (**Figure 3**). Soft tissues evaluated by MRI in the current study included masseter, temporal, digastric, pterygoid, and mylohyoid muscles for various affected ORN sites. For the sake of comparisons, the masseter and temporal muscles were considered the superficial muscle group, while the latter three were considered the deep muscle group. Preoperative trismus was classified according to Sakai’s criteria (13), with a mouth opening distance <10 mm as severe. Due to the varied ORN and fistula conditions, dual mastication and swallowing functions were reflected by preoperative food scale questionnaires. After obtaining these numbers, the multidimensions of STIs for mandibular ORN were preliminarily assessed.

### Cervical Vessel Assessment and Reconstructions

The cervical vessels were also evaluated by preoperative ultrasonic examination and intraoperative findings. Color duplex sonography (CDS), which provided data for vessel caliber, peak flow velocity (PV), and resistance index (RI), were used for analyses of the three branches of the external carotid artery, i.e., facial artery (FA), superior thyroid artery (STA), and lingual artery (LA).

In addition, the reconstructive approaches were summarized for both bone and soft tissue coverages. The soft-tissue defect sizes pre- or intraoperatively were measured and compared as indirect reflections of the fibrosis severity.

### Follow-Up and Functional Recovery

After debridement, the mouth-opening distances were regularly evaluated at the 6-month follow-up. Radiologically speaking, the reconstructed/resected mandibles were evaluated in a closed-mouth panoramic X-ray for both midline alignment and temporomandibular joint locations. Specifically, midline alignment was determined as the midpoint between the middle upper incisors. Improvement of mouth opening was defined as an increase in distance > 10 mm. In addition, the temporomandibular joint (TMJ) positions on the affected sides were also appraised. Facial contour was judged by comparing pre- and postoperative changes by the patients themselves: 0: no change; 1: slightly better, 2: much better, and 3: perfect. Mastication and swallowing functions were also assessed by the same food-scale questionnaire recorded preoperatively and at six months of follow-up by the Nutrition Rehabilitation Department. Speech intelligibility was measured by patients as “poor,” “good,” or “excellent” both pre- and postoperatively (at six months of follow-up). In addition, the quality of speech was also measured by using the classic conversational understandability test for objective evaluations (14). An audio recording of a 5-min conversation *via* telephone was evaluated by a group of three untrained normal student volunteers for conversational understandability using a 5-point scale: 5, all speech is understood; 4, sometimes not understood; 3, can be understood when conversational content is already known; 2, sometimes understood; and 1, nothing is understood. The STI factors were compared for their correlations with the multidimensional outcomes of ORN treatment (i.e., complications, recurrences, improvement of mouth opening/speech/mastication, midline alignment, facial contour changes).

### Statistical Analysis

Statistical analysis was performed using SPSS version 23.0 software (IBM Corp., Armonk, NY). Categorical or continuous variables were compared for the correlation between three ORN factors (predictors), i.e., ORN stages, radiation doses, sizes of ORN bones, and using logistic and linear regression, where appropriate. All the STI predictors were also compared with the parameters during postoperative follow-up. To decrease the confounding error (suppressor effect) caused by covariance and small sample size, multivariate correlation analysis was also performed, including the variables for which the p values of the univariate analysis were < 0.1. However, the final significance level for both univariate and multivariate analyses was still 0.05.

## Results

### Clinical and Demographic Information

A total of 47 patients with advanced mandibular ORN were included in the study. The median follow-up time was 27 months (range, 12 to 46 months). The patients’ general characteristics are shown in **Supplementary Table 3**. The median age at diagnosis was 56 years (range, 28–71 years), while 61.7% were male. Nasopharyngeal cancer was the histology type (from medical history) among most patients (n = 21, 44.7%), while those with oropharyngeal or oral cavity cancer comprised 14.9% (n = 7) and 27.7% (n = 13), respectively. Regarding prior treatment histories, a small number (n = 14, 29.8%) of patients received sequential or concurrent chemoradiation, while histories of local ablative surgeries were found in 48.9% (n = 23). The median irradiation doses reached 62 Gy (range, 40–110 Gy), with 12 patients (25.5%) receiving higher doses over 70 Gy. Conservative treatment, mostly HBO therapy, was administered to 10 patients (21.3%) before our surgical debridement, but in vain.

The dual radiation-induced effects of both hard- and soft-tissue damage were quite evident in these cases with advanced mandibular ORN. According to our BS staging system, the entire study population was subcategorized into patients with stage II disease (n = 16, 34.0%) and those with stage III disease (n = 31, 66.0%). Radiologically, most (n = 44, 93.5%) of the ORN lesions were found in the ipsilateral mandibular body and/or ramus. Detailed information on hard-tissue toxicity was also presented with the ORN mandibular bone sizes (average length: 7.9 cm). Most of the defects (n = 26, 55.3%) after segmental mandibulectomy and debridement were classified as type II according to Brown’s classification, within whom six (12.8%) had condylar removal as type IIc. After multivariate analysis, a positive correlation (p = 0.040) between dose and histology type was established. Due to the statistical significance (p = 0.007) revealed in **Supplementary Table 3**, the affected mandibular subsite was correlated with ORN stages. In addition, the ORN bone sizes were related to both histology types (p = 0.003) and Brown’s classification (p < 0.001).

### STI Assessment

As revealed in **Supplementary Table 4**, the symptom scoring found an average of 5.4 (range 1 to 12) in our series, indicative of the severity of the STI problems. Within these symptoms, specific attention was also given to a relatively high number of cases with severe trismus (n = 19, 40.4%) and intra- or extraoral fistulas (n = 26, 55.3%), reflecting aggravated soft-tissue fibrosis or infections around mandibular ORN lesions. Similarly, soft-tissue toxicity caused by irradiation was also found in most preoperative MRI examinations, as signals of hypertrophy or edema (n = 41, 87.2%) were frequently discerned in those with serious bone destruction, implying corresponding clinical discomfort in advanced ORN patients. The intraoperative findings of soft-tissue debridement revealed that the real STIs surrounding advanced ORN far exceeded the expected local mucosal involvement, as most patients (n = 23, 48.9%) had extensive muscular fibrosis and infection, which required further debridement, extensive scar release, or even coronoidectomy (in addition to local mandibulectomy), with the aim of fully resolving the complicated trismus caused by ORN. Another angle of investigation was also obtained, as such severe fibrosis (trismus) was also related to the amount of irradiation doses received by patients (p = 0.013). Surprisingly, although the current ORN stages were correlated with the ORN sizes found in CT scans (p = 0.045), the staging system did not reflect the STIs in these patients, as no significance was found in the multivariate statistical analyses. On the other hand, as shown in our statistics, most STI factors, except cervical fibrosis (p = 0.02), did not have a parallel tendency with the bone destruction levels of ORN. Contrary to our original belief of STI and ORN relations, even the intraoperative soft-tissue defect changes could not be indirectly extrapolated by the extent of ORN bone damage (p = 0.096).

### STI-Related Vessel Characteristics and Reconstructions

In consideration of the debridement and reconstruction designs, the relations between ORN-related factors were also compared with multidimensional data reflecting radiation-induced cervical vessel damage. Cervical vessel impairment, as another aspect of STIs, was measured for all three index arteries (FA, STA, and LA) for various degrees of angiostenosis or hemodynamic compromises (**Supplementary Table 5** **)**. Judging from the CDS results, the ipsilateral superficial arteries (FA) were more prone to be adversely affected by irradiation due to the increased rate of narrower calibers (caliber<1 mm or not found, n = 22, 46.8%) or slower blood flows (PV< 40 cm/s, n = 39, 83.0%). STA showed similar radiation-induced hemodynamic effects (PV<40 cm/s, n = 40, 85.2%), while the caliber was less affected due to a relatively smaller portion of sizes lower than 1 mm (n = 14, 28.9%). Accordingly, for easier intraoperative anastomosis, deeper vessels with better vascular qualities, such as STA (n = 22, 46.8%) and LA (n = 5, 10.6%), either on the ipsilateral or contralateral sides, were used in most circumstances. Fibular flaps (n = 33, 70.2%) were most frequently used in this study for functional mandibular repair, while pedicled pectoralis myocutaneous flaps (PMMFs) were also used for cases (n = 9, 19.1%) with severe fibrosis and unavailable vessels. All flaps survived despite two cases with successful postoperative management of venous crises. Within all the reconstructive factors, the relation between ORN stage and reconstruction was established after multivariate analysis. However, the extent of cervical vessel damage was not related to ORN factors, as none of the vascular measurement data could be speculated after simple observation of ORN lesions.

### Postoperative Follow-Up and Functional Evaluations

**Figure 4:**
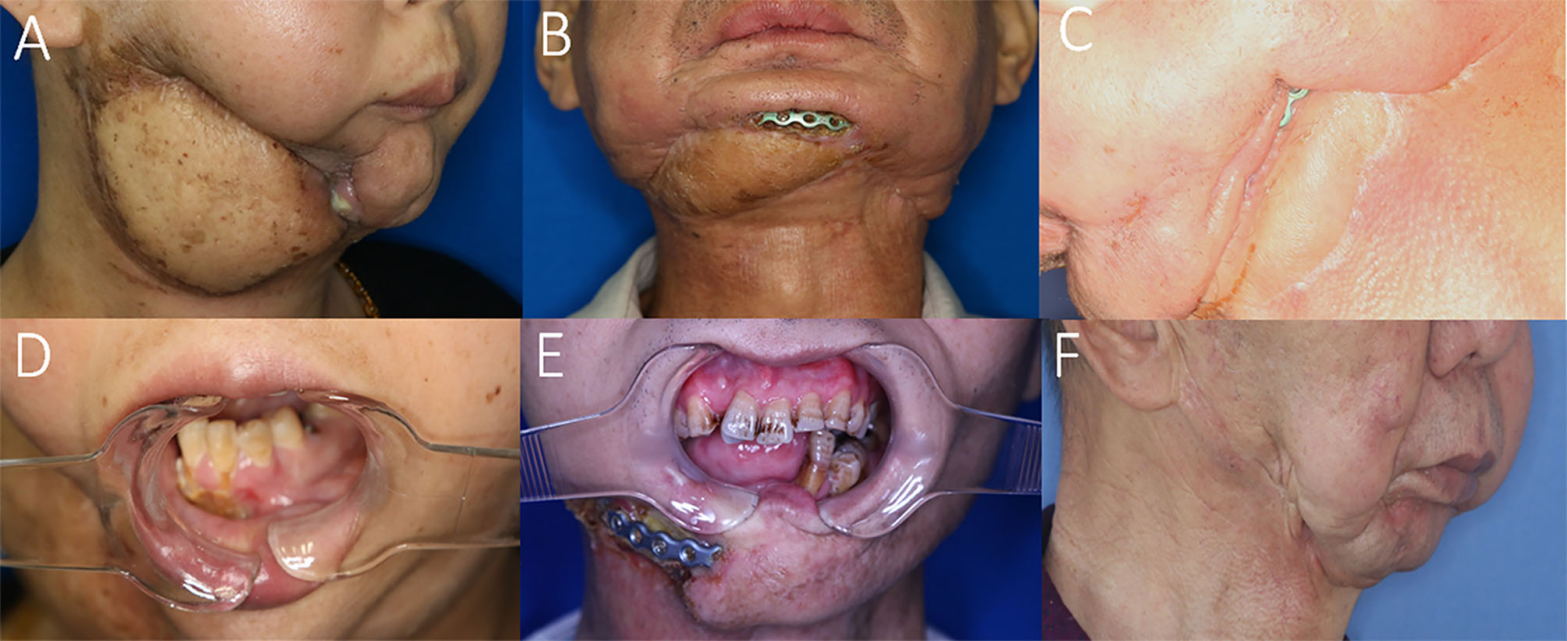
Representative cases with recurrences and complications possibly due to STI mismanagement. **(A)** Insufficient scar release and soft tissue debridement causing anterior bone exposure and oro-cutaneous fistula 2 months after ORN treatment. **(B)** Insufficient soft-tissue component for tissue coverage in the anterior mandibular region after ORN and STI debridement implying inconsiderate reconstructive design. **(C)** Insufficient soft-tissue coverage causing plate exposure in the mandibular angle region. **(D)** The same patient of A with postoperative unrelieved trismus despite ORN mandibulectomy. **(E)** Postoperative trismus and recurrence of ORN due to both insufficient bone and soft-tissue management. **(F)** Undesirable facial contour change and midline misalignment in the left-sided concaved lower face, due to erroneous scar release and insufficient soft tissue flap coverage.

The major complications after surgical debridement (with/without reconstructions) were lung infection (n = 4, 8.5%), wound dehiscence (n = 4, 8.5%) and fistula (n = 2, 4.3%), with representative cases shown in **Figure 4**. For the short-term outcomes of our surgical treatment, a relatively low recurrence rate (n = 3, 6.9%) was reported during the follow-up. Apart from recurrences, other dimensions of surgical debridement revealed that trismus symptoms were ameliorated in 19 patients (40.4%), while a moderate number (n = 30, 53.8%) of patients had relatively favorable (≤10 mm) midline alignment after debridement. However, for the other cases in this cohort the symptom relief improvement was not evident. A similar trend was also observed from the subjective review of the facial contour changes (aesthetics) by the patients. On the other hand, the outcomes of functional recovery were mediocre due to the dichotomized evaluations of both mastication/swallowing and conversation, as great improvement was only found in 19 (35.2%) and 8 (17.0%) respectively (**Supplementary Table 6**).

The statistical analyses showed a possible correlation (p = 0.016) between ORN hard-tissue injuries and postoperative midline alignment. In addition, a pronounced difference (p = 0.041) was also discerned between radiation dose and postoperative facial changes in the univariate analysis, notwithstanding the negative (p = 0.054) multivariate result. In other outcome analyses, the correlations between ORN factors and functional (aesthetic) or symptom relief outcomes were mostly not significant (**Supplementary Table 6**).

### STI Factors and Their Relations With Treatment Outcomes

On the other hand, for the STI-related factors, we found a strong correlation (p = 0.004) between the STI symptom score and recurrence (**Supplementary Table 7**, representative cases in **Figure 4**). The detailed analyses of the STI factors revealed a general trend towards ORN recurrences in those with more severe STIs, such as trismus, soft-tissue debridement, MRI evaluations of edema and cervical fibrosis with vessel stenosis, although most p values did not reach significance due to the small sample size of recurrences. In addition, when taking trismus into consideration, both the improvement in mouth-opening distance (p<0.001) and facial contour change (p = 0.004) were adversely affected, as corroborated in our statistics, indicative of refractory fibrosis unchanged by simple bone debridement or limited STI management. In addition, the improvement in mouth-opening distance was also influenced by signals of muscle group involvement in the MRI scans (p = 0.028), which implied the status of preoperative soft-tissue fibrosis and the difficulty of management for both hard- and soft-tissue problems in some late-staged ORN cases. In addition, a correlation was observed as well between intraoperative soft-tissue defect changes and development of postoperative complications (p = 0.024), despite the low complication rate in this cohort. A significant relationship (p = 0.012) was also found between facial contour change and intraoperative soft-tissue defect changes, highlighting the considerations of potential long-lasting detrimental effects of radio-induced STIs.

### Assessment of STIs and Proposed Algorithm for Soft-Tissue Considerations and Management in ORN Cases

**Figure 5:**
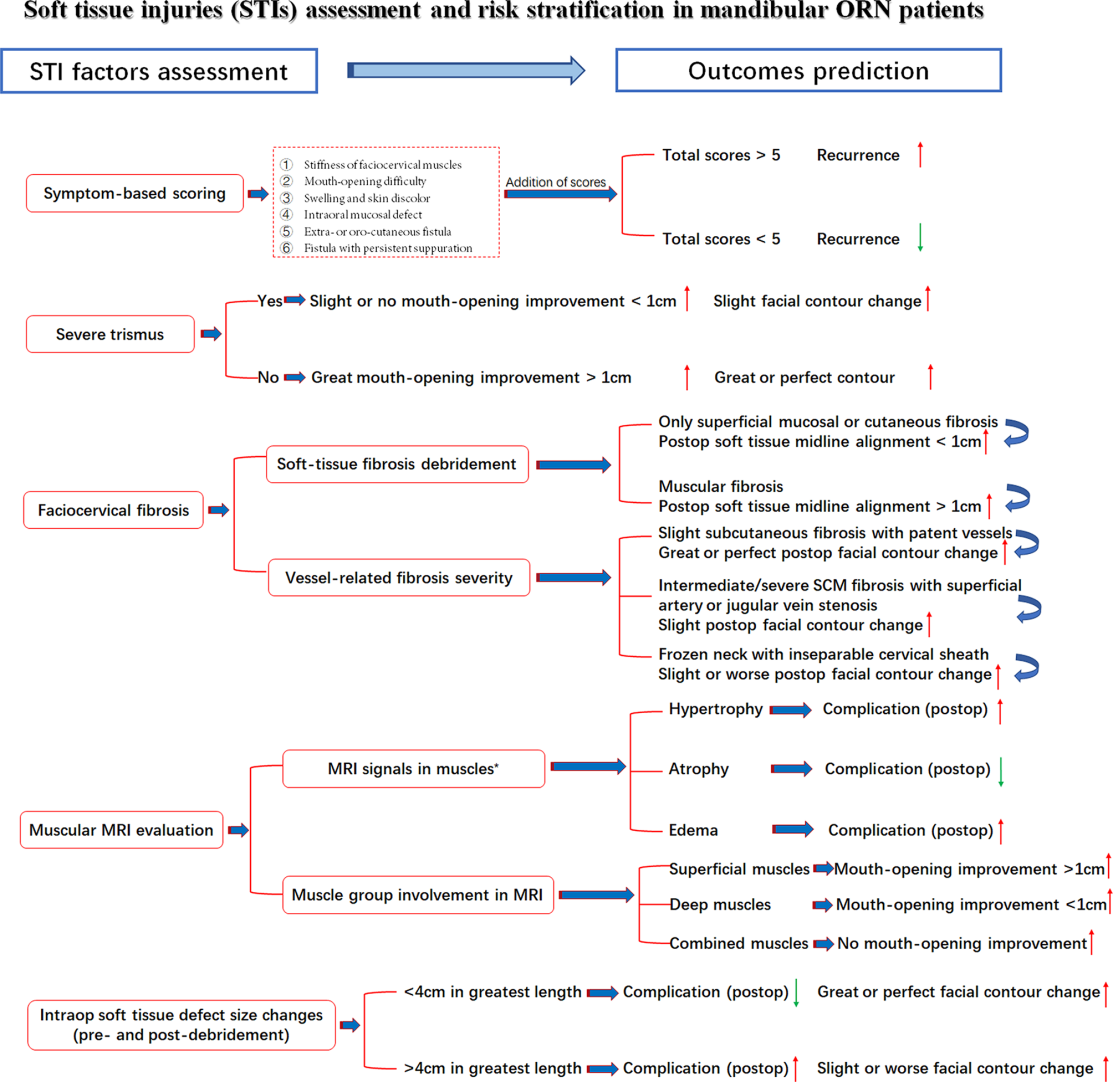
STI assessment with seven related factors, risk stratifications and outcome prediction. Red arrow: Higher/increased probability of outcomes; Green arrow, Lower/decreased probability of outcomes; *, Observed tendency despite insignificant p-value.

Under the aforementioned confirmation of STI influences on treatment outcomes, the comprehensive assessment of preoperative STI in mandibular ORN cases is presented in **Figure 5**. The risk stratification of multifactorial STIs and their possible specific relations with outcomes were also included, with red arrows indicating increased likelihood and green for decreased one.

**Figure 6:**
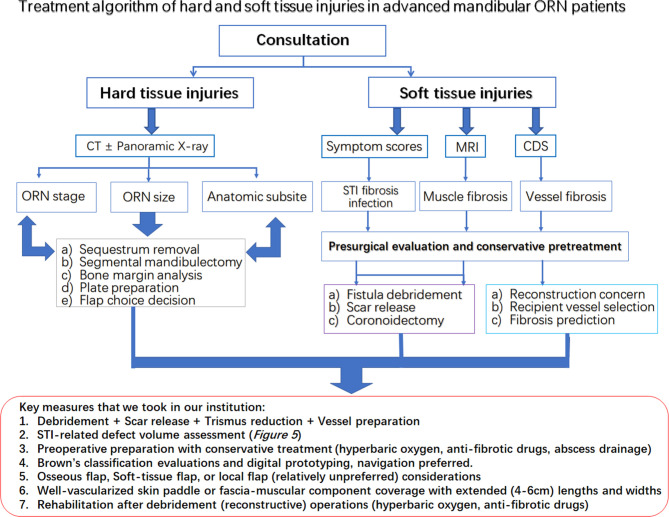
The treatment algorithm for hard and soft tissue injuries in the advanced mandibular ORN patients. Red rectangular frame: The key measures taken in our institution for both bone and STI management.

In addition, based on these results, we tentatively proposed an algorithm combining evaluations, presurgical STI evaluation, presurgical preparation, surgical designs, and postoperative functional predictions for STIs in advanced mandibular ORN (**Figure 6**). The experiences of ORN and STI treatment were shared for key measures during the whole process of design, preparation and operations. Preliminary considerations were also summarized in this algorithm as well.

## Discussion

Although irradiation of bone is a prerequisite for the development of ORN, STIs, unlike bone lesions, STI have not been given sufficient attention (15, 16). The STIs surrounding ORN lesions always manifest themselves as late (long-term) toxicities of radiation, resulting in “radiation fibrosis syndrome” with progressive functional losses (17). Although the prevalence of common radiation-induced STIs, such as trismus, dysphagia, and xerostomia, varies from 21% to 75% depending on specific anatomic subsites, tumor histology, and treatment regimens, the conditions of soft tissues in ORN patients remain largely unknown (18–20). Such negligence of soft-tissue toxicity has caused wide confusion during the presurgical evaluation process, thereby resulting in various ORN treatment outcomes (21–23). Despite a general trend towards aggressive surgical approaches for mandibular ORN, surrounding STIs were treated conservatively with inclinations of limited debridement or simple fistula repairs in the literature (23–25). Moreover, key soft tissue debridement points were not mentioned in some articles. Clinically speaking, sequestrum removal or mandibular resection in ORN patients is relatively simple (26), while the challenge of surgical management, as we perceive, rests mostly on the long-term detrimental changes caused by irradiation-induced STIs. First, the profound and irreversible consequences caused by irradiation will, in theory, result in substantial dermal, epidermal, or even muscular induration, scarring, and retractions (27). The anatomic plane would be greatly blurred, subsequently causing difficulty in surgical assessment. In addition, unlike bone, soft-tissue margins were harder to find in ORN cases for severe fibroses and infections (23, 28, 29). In addition, sufficient well-vascularized soft-tissue flaps were sometimes mandatory for the coverage of such defects; otherwise, unfavorable wound healing would ensue (23), as revealed in our cases. Another undesirable outcome in the treatment of ORN patients was unrelieved trismus, which was also not mentioned in many studies. In addition to hard tissue-oriented factors, such as osteolysis or fracture, trismus in ORN patients was partially generated due to an underestimation of muscular or ligament fibrosis, which would also constrain TMJ movement (30, 31). Apart from these, from a surgical perspective, the severity of radiation-induced vascular damage would sway the decisions for post-ablative ORN reconstructions (32). The relations between the availability, or more precisely the quality, of the existing vessels and the extent of ORN were not well established. We found that the ORN bone severity and vascular parameters were mostly irrelevant, as no significance was found between these variables in our study. Cervical vessel damage, as far as we are concerned, is more or less affected by triplex factors including cervical radiation doses, prior treatment history and infectious severity. Firstly, the discrepancies between the doses on mandible and neck varied in different patients concerning various disease pathologies and clinical stages. Second, some of our patients in this study received prior head and neck operations, which might also aggravate the fibrosis and STI damages to the cervical vessels. On the other hand, due to different infectious status of patients (evaluated at admission), locoregional vessels might be partially influenced due to the long-term tissue swelling and accompanying infection-related fibrosis. Thus, due to irrelevance between ORN bone severity and cervical vessel damages, the evaluation of cervical vessels entails further examinations, such as CDS or CTA (33), which was also frequently used in our cases.

Based on our statistics, we advocate that along with nonviable sequestrum removal, the successful debridement of local ORN lesions also entails a full grasp of the information regarding scar release, infection control, vessel confirmation, and fibrotic muscle resections. Such considerations or measures, though occasionally mentioned in some ORN staging systems, have not been clearly summarized (34–36). Based on Marx’s 3-stage system, advanced ORN cases were categorized only for the patients’ responsiveness to HBO treatment (34). From a surgical standpoint, Notani first introduced a classification of mandibular ORN on the severity of osteolysis. However, such classification was solely based on the depths of hard-tissue involvement (alveolar, above or beyond alveolar canal invasions), with a lack of STI evaluations (35). Although our previous BS staging system was refined to incorporate evaluations of both radiological and clinical manifestations, the only and rough assessment of soft-tissue fistula is still not comprehensive (10). The inherent loopholes in the BS system were quite obvious, as most soft-tissue problems mentioned in this study, such as trismus, muscular breakdown, and vascular stenoses, were largely undetermined. Thus, according to our new treatment algorithm against STIs in advanced ORN, comprehensive soft-tissue assessment procedures were introduced, while STI factors in ORN patients were also demonstrated for the first time. The management centered around the STIs, as an amendment, was updated to our BS staging system, with concerns for better functional outcomes.

Most surgeons were often bewildered at the conundrums of whether ORN and STI had reciprocal relations and how these relations were influenced by different variables. Unfortunately, despite the extensive current studies on radio-induced bone damage, the ORN and STI associations, specifically soft-tissue evaluations, especially in advanced ORN cases, have not been elucidated, either on an etiological or therapeutic level (37, 38). As in our studies, dose, consistent with other STI studies, was also correlated with the severity of trismus in advanced ORN patients (18, 30). However, most STIs, except cervical fibrosis, were not in parallel with the bone destruction levels of ORN. This finding pointed out the relatively independent role of STI evaluations in ORN patients while alarming the necessity of enhanced efforts for STI management. Within all the STI variables, scarring symptoms, such as trismus and cervical fibrosis, were both associated with the surgical outcomes. Intraoperative soft-tissue defect changes could also, to some extent, influence the restoration of facial esthetic and speech functions. In addition, there exists moderate evidence that the lasting superimposition of trismus, fibrosis, or other soft-tissue toxicities will contribute to an increased deterioration of overall functions and esthetics of head and neck cancer survivors (5, 8, 39, 40). Such a phenomenon was also observed in our cases, as the severity of multifactorial STIs would adversely affect the incidence of surgical complications, the improvement of trismus, or even mastication and swallowing functions. In this sense, despite vigorous attempts at more precise mandibular reconstructions (continuity, midline alignment, and TMJ positions), the significance of resolving soft tissue concerns should never be underrated. Thus, we came up with the first risk stratifications of these STI-related factors in mandibular ORN patients. Detailed assessment information was presented in **Figure 5** for possible outcome prediction.

Within our management algorithm for STI evaluations, a complete workup or consultation should be first applied to patients with advanced ORN. All hard tissue-related factors, such as prior treatment history and irradiation dose, should be clearly recorded for ORN hard-tissue management. During the medical consultation, a regular interview highlighting the STI-related soft tissue burdens should also be recorded (41). First of all, the STI-related symptoms should be recorded for risk stratification of ORN recurrence, as proved in our study. Trismus severity, as a core element of STIs, should be measured with clinical examinations in ORN patients, which is consistent with other radiation-induced toxicity reports (3, 13). Other symptoms, such as stiffness of local muscles, should be preliminarily assessed by facial or cervical tightness during mastication or head rotation for a preoperative impression of the extent of ORN-oriented fibrosis (42). In addition, precautions of vessel insufficiency or large skin defects, as revealed in our analysis, could be guarded in those with such symptoms. Radiographically speaking, for further well-round STI assessment, along with CT scans, MRI and CDS are mandatory for the assessment of surrounding muscles and vessels (43). MRI examinations could reveal the types of soft-tissue involvement in ORN cases, implying additional muscle resections or scar release when signals of “hypertrophy” and “edema” were shown in surrounding muscles (12). The CDS should always been used out of a reconstructive concern for finding the most suitable vessels during the preoperative assessment, since cervical vessel status was not related to radiation dose or ORN bone severity according to our study. For patients with severe fibrosis and trismus, preoperative conservative measures, such as antibiotic, hyperbaric oxygen (HBO) and anti-fibrotic drugs [pentoxifylline-tocopherol-clodronate (PENTOC)] can, in our opinion, be applied in selected patients for ameliorating the STI related damages. We figure that the first priority during the debridement operation should be given to finding viable anastomosis vessels, releasing soft-tissue fibrosis for exposure, and always prepared for coronoidectomy due to trismus relief concern. Due to the possible correlation established in the present study between intraoperative defect size changes and postoperative functional recovery, we also advocated designs of oversized and well-vascularized soft-tissue skin paddles or components for sufficient wound coverage after STI debridement in advanced ORN cases (44, 45). Sometimes, it is not wise to attain osseous mandibular reconstructions in the first attempt when STIs accompanying ORN are severe. The occlusion can be safely maintained with removable gap-keeping protheses for a possible secondary osseous bone reconstruction. In addition, when it comes to soft tissue debridement, we are always inclined to err on the safe side for being a bit more aggressive in treating the STIs in advanced ORN cases. The fibrotic tissues, surrounding ORN bone lesions, should be checked for both vascularity and elasticity. Some soft tissues, especially those with radioinduced or infectious stiffness texture tend to cause serious wound dehiscence even when a large bulky flap was utilized. The causes of such complications, as far as we are concerned, are due to the postoperative tissue retraction and insufficient subcutaneous scar release, both leading to local deficiency of subdermal circulations. Besides, postoperative reinforcement measures, such as HBO or PENTOC, are also advocated for increasing local tissue viability (**Figure 6**).

Lastly, this work has limitations due to the retrospective design, and the data were from a single institution only. In addition, the number of patients with advanced ORN was relatively small. Some of the evaluations were also subjective owing to the varied histories and conditions of ORN patients. Admittedly, our findings need to be viewed with caution pending a larger multi-institutional study, but the data can help to guide decisions and prognoses about the treatment of individual ORN patients with severe STI burdens.

## Conclusions

The coexistence of various hard- and soft-tissue damages in advanced mandibular ORN patients reminds surgeons of significance in comprehensive assessment of the dual aspects. It is necessary to take the same active measures to repair severe STIs as those for ORN bone lesions. For better functional outcomes, STI factors should always be considered during the ORN treatment process.

## Data Availability Statement

The raw data supporting the conclusions of this article will be made available by the authors, without undue reservation.

## Ethics Statement

The studies involving human participants were reviewed and approved by the Independent Ethics Committee of Shanghai Ninth People’s Hospital, Shanghai Jiao Tong University School of Medicine. The patients/participants provided their written informed consent to participate in this study. Written informed consent was obtained from the individual(s) for the publication of any potentially identifiable images or data included in this article.

## Author Contributions

YH provided the idea for this study and designed the algorithm. XL and DZ collected the clinical and radiological data, while CM wrote the manuscript. WG performed the statistical analysis and aligned the tables and figures for this study. FZ and ZL collected the follow-up functional data and revised the manuscript. All authors contributed to the article and approved the submitted version.

## Funding

This study was supported by Science and Technology Commission of Shanghai Municipality, grant 19ZR1430000; Hospital Innovation Project, grant CK2019004; Hospital Cross-Multidisciplinary Project, grant JYJC201911.

## Conflict of Interest

The authors declare that the research was conducted in the absence of any commercial or financial relationships that could be construed as a potential conflict of interest.

## References

[1] De Felice F Tombolini V Musio D Polimeni A. Radiation therapy and mandibular osteoradionecrosis: State of the art. Curr Oncol Rep (2020) 22(9):89. 10.1007/s11912-020-00954-3 32642937

[2] Johnson DE Burtness B Leemans CR Lui VWY Bauman JE Grandis JR. Head and neck squamous cell carcinoma. Nat Rev Dis Primers (2020) 6(1):92. 10.1038/s41572-020-00224-3 33243986 PMC7944998

[3] Sroussi HY Epstein JB Bensadoun RJ Saunders DP Lalla RV Migliorati CA. Common oral complications of head and neck cancer radiation therapy: mucositis, infections, saliva change, fibrosis, sensory dysfunctions, dental caries, periodontal disease, and osteoradionecrosis. Cancer Med (2017) 6(12):2918–31. 10.1002/cam4.1221 PMC5727249 29071801

[4] Madrid C Abarca M Bouferrache K. Osteoradionecrosis: An update. Oral Oncol (2010) 46(6):471–4. 10.1016/j.oraloncology.2010.03.017 20457536

[5] Lee M Chin RY Eslick GD Sritharan N Paramaesvaran S. Outcomes of microvascular free flap reconstruction for mandibular osteoradionecrosis: A systematic review. J Craniomaxillofac Surg (2015) 43(10):2026–33. 10.1016/j.jcms.2015.03.006 26427619

[6] Jacobson AS Buchbinder D Hu K Urken ML. Paradigm shifts in the management of osteoradionecrosis of the mandible. Oral Oncol (2010) 46(11):795–801. 10.1016/j.oraloncology.2010.08.007 20843728

[7] He Y Ma C Hou J Li X Peng X Wang H. Chinese expert group consensus on diagnosis and clinical management of osteoradionecrosis of the mandible. Int J Oral Maxillofac Surg (2020) 49(3):411–9. 10.1016/j.ijom.2019.06.015 31353174

[8] Wong ATT Lai SY Gunn GB Beadle BM Fuller CD Barrow MP. Symptom burden and dysphagia associated with osteoradionecrosis in long-term oropharynx cancer survivors: A cohort analysis. Oral Oncol (2017) 66:75–80. 10.1016/j.oraloncology.2017.01.006 28249651 PMC5336132

[9] Dhanda J Pasquier D Newman L Shaw R. Current Concepts in Osteoradionecrosis after Head and Neck Radiotherapy. Clin Oncol (R Coll Radiol) (2016) 28(7):459–66. 10.1016/j.clon.2016.03.002 27038708

[10] He Y Liu Z Tian Z Dai T Qiu W Zhang Z. Retrospective analysis of osteoradionecrosis of the mandible: proposing a novel clinical classification and staging system. Int J Oral Maxillofac Surg (2015) 44(12):1547–57. 10.1016/j.ijom.2015.04.006 26169162

[11] Brown JS Barry C Ho M Shaw R. A new classification for mandibular defects after oncological resection. Lancet Oncol (2016) 17(1):e23–30. 10.1016/S1470-2045(15)00310-1 26758757

[12] Van de Meent MM Pichardo SEC Rodrigues MF Verbist BM Van Merkesteyn JPR. Radiographic characteristics of chronic diffuse sclerosing osteomyelitis/tendoperiostitis of the mandible: A comparison with chronic suppurative osteomyelitis and osteoradionecrosis. J Craniomaxillofac Surg (2018) 46(9):1631–6. 10.1016/j.jcms.2018.06.015 30017711

[13] Dijkstra PU Huisman PM Roodenburg JL. Criteria for trismus in head and neck oncology. Int J Oral Maxillofac Surg (2006) 35(4):337–42. 10.1016/j.ijom.2005.08.001 16280237

[14] Taguchi T. Speech evaluation. In: Taguchi T, editor. Treatment of Speech Disorder. Tokyo: Igakushoin (1966). p. 37–8.

[15] Tsai CJ Hofstede TM Sturgis EM Garden AS Lindberg ME Wei Q. Osteoradionecrosis and radiation dose to the mandible in patients with oropharyngeal cancer. Int J Radiat Oncol Biol Phys (2013) 85(2):415–20. 10.1016/j.ijrobp.2012.05.032 22795804

[16] Chopra S Kamdar D Ugur OE Chen G Peshek B Marunick M. Factors predictive of severity of osteoradionecrosis of the mandible. Head Neck (2011) 33(11):1600–5. 10.1002/hed.21654 21484922

[17] Delanian S Lefaix JL. Current management for late normal tissue injury: Radiation-induced fibrosis and necrosis. Semin Radiat Oncol (2007) 17(2):99–107. 10.1016/j.semradonc.2006.11.006 17395040

[18] Gebre-Medhin M Haghanegi M Robért L Kjellén E Nilsson P. Dose-volume analysis of radiation-induced trismus in head and neck cancer patients. Acta Oncol (2016) 55(11):1313–7. 10.1080/0284186X.2016.1221129 27595312

[19] Strojan P Hutcheson KA Eisbruch A Beitler JJ Langendijk JA Lee AWM. Treatment of late sequelae after radiotherapy for head and neck cancer. Cancer Treat Rev (2017) 59:79–92. 10.1016/j.ctrv.2017.07.003 28759822 PMC5902026

[20] Rogus-Pulia NM Pierce MC Mittal BB Zecker SG Logemann JA. Changes in swallowing physiology and patient perception of swallowing function following chemoradiation for head and neck cancer. Dysphagia (2014) 29(2):223–33. 10.1007/s00455-013-9500-y 24402239

[21] Rogers SN D’Souza JJ Lowe D Kanatas A. Longitudinal evaluation of health-related quality of life after osteoradionecrosis of the mandible. Br J Oral Maxillofac Surg (2015) 53(9):854–7. 10.1016/j.bjoms.2015.07.008 26316016

[22] Wang L Su YX Liao GQ. Quality of life in osteoradionecrosis patients after mandible primary reconstruction with free fibula flap. Oral Surg Oral Med Oral Pathol Oral Radiol Endod (2009) 108(2):162–8. 10.1016/j.tripleo.2009.03.005 19464214

[23] Suh JD Blackwell KE Sercarz JA Cohen M Liu JH Tang CG. Disease relapse after segmental resection and free flap reconstruction for mandibular osteoradionecrosis. Otolaryngol Head Neck Surg (2010) 142(4):586–91. 10.1016/j.otohns.2009.12.008 20304283

[24] Manzon L Rossi E Fratto G. Management of osteonecrosis of the jaws induced by radiotherapy in oncological patients: preliminary results. Eur Rev Med Pharmacol Sci (2015) 19(2):194–200. 25683930

[25] Hirsch DL Bell RB Dierks EJ Potter JK Potter BE. Analysis of microvascular free flaps for reconstruction of advanced mandibular osteoradionecrosis: a retrospective cohort study. J Oral Maxillofac Surg (2008) 66(12):2545–56. 10.1016/j.joms.2007.08.041 19022135

[26] Cannady SB Dean N Kroeker A Albert TA Rosenthal EL Wax MK. Free flap reconstruction for osteoradionecrosis of the jaws–outcomes and predictive factors for success. Head Neck (2011) 33(3):424–8. 10.1002/hed.21463 20645290

[27] Borrelli MR Shen AH Lee GK Momeni A Longaker MT Wan DC. Radiation-induced skin fibrosis: pathogenesis, current treatment options, and emerging therapeutics. Ann Plast Surg (2019) 83(4S Suppl 1):S59–64. 10.1097/SAP.0000000000002098 PMC6746243 31513068

[28] Rommel N Kesting MR Rohleder NH Wolff KD Weitz J. Surgical management of severe osteoradionecrosis of the mandibular bone by using double free flap reconstruction. J Craniomaxillofac Surg (2018) 46(1):148–54. 10.1016/j.jcms.2017.09.025 29174551

[29] O’Dell K Sinha U. Osteoradionecrosis. Oral Maxillofac Surg Clin North Am (2011) 23(3):455–64. 10.1016/j.coms.2011.04.011 21798443

[30] van der Geer SJ Kamstra JI Roodenburg JL van Leeuwen M Reintsema H Langendijk JA. Predictors for trismus in patients receiving radiotherapy. Acta Oncol (2016) 55(11):1318–23. 10.1080/0284186X.2016.1223341 27627138

[31] Kraaijenga SA Hamming-Vrieze O Verheijen S Lamers E van der Molen L Hilgers FJ. Radiation dose to the masseter and medial pterygoid muscle in relation to trismus after chemoradiotherapy for advanced head and neck cancer. Head Neck (2019) 41(5):1387–94. 10.1002/hed.25573 30652390

[32] Tall J Björklund TC Skogh AC Arnander C Halle M. Vascular complications after radiotherapy in head and neck free flap reconstruction: Clinical outcome related to vascular biology. Ann Plast Surg (2015) 75(3):309–15. 10.1097/SAP.0000000000000081 25003403

[33] Thankappan K. Microvascular free tissue transfer after prior radiotherapy in head and neck reconstruction - A review. Surg Oncol (2010) 19(4):227–34. 10.1016/j.suronc.2009.06.001 19570672

[34] Lyons A Osher J Warner E Kumar R Brennan PA. Osteoradionecrosis—a review of current concepts in defining the extent of the disease and a new classification proposal. Br J Oral Maxillofac Surg (2014) 52(May(5):392–5. 10.1016/j.bjoms.2014.02.017 24725905

[35] Notani K Yamazaki Y Kitada H Sakakibara N Fukuda H Omori K. Management of mandibular osteoradionecrosis corresponding to the severity of osteoradionecrosis and the method of radiotherapy. Head Neck (2003) 25(3):181–6. 10.1002/hed.10171 12599284

[36] Schwartz HC Kagan AR. Osteoradionecrosis of the mandible: Scientific basis for clinical staging. Am J Clin Oncol (2002) 25(2):168–71. 10.1097/00000421-200204000-00013 11943896

[37] Qaisi M Montague L. Bone Margin Analysis for Osteonecrosis and Osteomyelitis of the Jaws. Oral Maxillofac Surg Clin North Am (2017) 29(3):301–13. 10.1016/j.coms.2017.03.007 28709531

[38] Cheriex KC Nijhuis TH Mureau MA. Osteoradionecrosis of the jaws: A review of conservative and surgical treatment options. J Reconstr Microsurg (2013) 29(2):69–75. 10.1055/s-0032-1329923 23258622

[39] Stubblefield MD. Radiation fibrosis syndrome: neuromuscular and musculoskeletal complications in cancer survivors. PM R (2011) 3(11):1041–54. 10.1016/j.pmrj.2011.08.535 22108231

[40] Stubblefield MD. Clinical Evaluation and Management of Radiation Fibrosis Syndrome. Phys Med Rehabil Clin N Am (2017) 28(1):89–100. 10.1016/j.pmr.2016.08.003 27913002

[41] Hamilton SN Arshad O Kwok J Tran E Fuchsia Howard A Serrano I. Documentation and incidence of late effects and screening recommendations for adolescent and young adult head and neck cancer survivors treated with radiotherapy. Support Care Cancer (2019) 27(7):2609–16. 10.1007/s00520-018-4559-5 30467794

[42] Ortiz-Comino L Fernández-Lao C Speksnijder CM Lozano-Lozano M Tovar-Martín I Arroyo-Morales M. Upper body motor function and swallowing impairments and its association in survivors of head and neck cancer: A cross-sectional study. PLoS One (2020) 15(6):e0234467. 10.1371/journal.pone.0234467 32559241 PMC7304609

[43] Deshpande SS Thakur MH Dholam K Mahajan A Arya S Juvekar S. Osteoradionecrosis of the mandible: through a radiologist’s eyes. Clin Radiol (2015) 70(2):197–205. 10.1016/j.crad.2014.09.012 25446325

[44] Danielsson D Gahm C Haghdoost S Munck-Wikland E Halle M. Osteoradionecrosis, an increasing indication for microvascular head and neck reconstruction. Int J Oral Maxillofac Surg (2020) 49(1):1–6. 10.1016/j.ijom.2019.06.009 31296436

[45] Bettoni J Olivetto M Duisit J Caula A Testelin S Dakpé S. The value of reconstructive surgery in the management of refractory jaw osteoradionecrosis: a single-center 10-year experience. Int J Oral Maxillofac Surg (2019) 48(11):1398–404. 10.1016/j.ijom.2019.06.007 31227272

